# Electromagnetic Detection System with Magnetic Dipole Source for Near-Surface Detection

**DOI:** 10.3390/s23249771

**Published:** 2023-12-12

**Authors:** Xian Liao, Zhengyu Xu, Wei Liu, Heng-Ming Tai, Jie Zhou, Xiao Ma, Zhihong Fu

**Affiliations:** 1 School of Electrical Engineering, Chongqing University, Chongqing 400044, China; 20124043@cqu.edu.cn (X.L.); 20221101035@stu.cqu.edu.cn (J.Z.); 20163690@cqu.edu.cn (X.M.); fuzhihong@cqu.edu.cn (Z.F.); 2 State Grid Chongqing Electric Power Company Electric Power Research Institute, Chongqing 401121, China; liuwei_cqu@163.com; 3 Department of Electrical and Computer Engineering, University of Tulsa, Tulsa, OK 74104, USA; tai@utulsa.edu

**Keywords:** transient electromagnetic (TEM), near-surface detection, BCPPS, magnetic dipole source

## Abstract

This paper proposes a nondestructive, separate transmitter-receiver (TX-RX) electromagnetic measurement system for near-surface detection. Different from the traditional dual-coil integrated design, the proposed transient electromagnetic (TEM) system performs shallow subsurface detection using independent TX coil and movable RX coils. This configuration requires a large primary field so that the far-away secondary field is able to generate reliably induced voltages. To achieve this goal, a bipolar current-pulsed power supply (BCPPS) with a late resonant charging strategy is designed to produce a sufficiently large magnetic moment for the exciting coil with low source interference. The magnetic dipole source (MDS) with a large proportion of weight is separated from the field observation device and does not need to be dragged or transported during the detection process. This setup lowers the weight of the scanning device to 3 kg and greatly improves the measurement efficiency. The results of the laboratory test verify the effectiveness of the separate MDS and RX module system. Field experimental detection further demonstrates that the proposed system can realize highly efficient and shallow surface detection within a 200 m range of the MDS device.

## 1. Introduction

Nondestructive detection using electromagnetic wave propagation characteristics for near-surface anomalies has received a lot of attention in different applications such as for geoarchaeological mapping, municipal maintenance, substation grounding grid detection, and unexploded bomb detection [1,2,3,4]. The time-domain transient electromagnetic method (TEM) typically employs two different multi-turn coils: the TX coil in the transmitter and the RX coil in the receiver. The former transmits the time-varying current to generate the primary electromagnetic field and the latter receives the secondary field-induced voltage generated from the eddy currents of subsurface conductors [5,6]. TEM systems are very effective in geological detection [7,8].

Conventional TEM detection devices configure the TX coils and RX coils in the same place [9]. The weak coupling design for the towed TEM system [10] improves detection sensitivity and the investigation depth; however, the calibration demands huge computational power. Moreover, it is difficult to accurately locate the relative position of the TX loop and RX coil. The opposing-coils TEM system [11,12] can reduce the effect of inherent mutual induction between the TX loop and RX coil at the expense of an increase in weight due to the complex coil configuration. The detection result is also sensitive to the vertical position of the RX coil. The towed TEM system uses the dual transmitter moment measurement technique to obtain geoelectric information on different subsurface layers [13]. The over-long offset (>9 m) between the TX loop and RX coil makes it difficult to operate the all-terrain vehicle and obtain valid data consistently. In frequency-domain detection, heavy or large-volume mobile scanning devices also cause implementation challenges, which greatly reduce detection efficiency and increase the cost [14,15].

Recently, airborne TEM (ATEM) systems have been developed for fast and convenient detection [16]. ATEM requires a large TX magnetic moment. Thus, TEM systems carried by helicopters utilize a gigantic coil with a radius of more than 20 m for deep geological surveys [17,18]. A bucking coil is connected on one side of the TX coil loop to achieve primary field compensation [19]. A transmitter with more electronic components and energy-storage devices is also needed to ensure that the current pulses in the coil can generate a strong primary field [20]. These TX-RX-integrated systems are not used for near-surface exploration due to their high cost and difficulty in operation.

For low-cost and large-region exploration, a semi-ATEM (SATEM) system, which uses a grounded-wire source on the surface to generate an electromagnetic field and a receiver hanging on unmanned aerial vehicles to acquire the secondary field in the air, has been developed [21,22]. This requires not only a solid electrical connection with the soil using electrodes or steel rods but also wires several kilometers long to send current pulses into the ground. The power supply also needs to provide tens of kW of power to ensure a sufficiently large current to excite the magnetic and electric fields due to high earth resistance and long grounded wires [23,24]. Moreover, the combined effects of the transmitter wire and grounding points challenge the simplicity of the secondary response [25].

In this paper, a fast electromagnetic measurement system for near-surface detection is proposed. Unlike current commercial TEM instruments, the fixed-position TX module and the mobile RX device are completely separated. Moreover, it has a similar detection mode to SATEM but uses a smaller magnetic source and is supplied with a lower power grade. The transmit coil in the proposed system employs a magnetic dipole source that is designed to circumvent the challenges associated with SATEM that require laying extensive transmit loops over several kilometers. Additionally, this system integrates a drone-mounted receiving system weighing a mere 3 kg, thereby sidestepping the logistical constraints of take-off sites and construction environments faced by manned helicopters. Concurrently, this drone-mounted approach significantly enhances detection efficiency compared to traditional ground-based TEM systems. A bipolar current pulse power supply (BCPPS) has been designed to overcome the magnetic field attenuation caused by the overlong offset between the transmitter and receiver. BCPPS is equipped with resonant charging in the late turn-off period to attain a large TX magnetic moment for the relatively small coil. With BCPPS and a high-precision sampling design, the proposed system can realize rapid scanning and the real-time detection of the near-surface within 200 m around the magnetic dipole source (MDS). In addition, the weight of the scanning unit is lowered to 3 kg, which greatly improves the construction efficiency and cuts the vehicle cost. The working principles, system design, and performance evaluation in the field experiment are elaborated in detail in the following sections.

## 2. Detection Principles

### 2.1. Magnetic Dipole Source for Rapid TEM Detection

**Figure 1:**
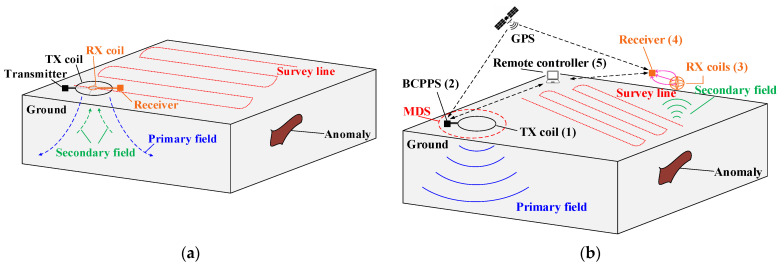
Schematics of the different TEM detection systems: (**a**) traditional TX-RX-integrated detection system; (**b**) proposed detection system with magnetic dipole source.

The TX coil and RX coil of TEM detection systems are usually integrated together and are arranged at the same central point [10,11,13,14]. Figure 1a depicts such a configuration. The offset between the RX coil and TX coil is either small or they are arranged at the same central point. In this configuration, a transmitter with a small magnetic moment output can ensure the magnitude of the induced voltages; however, the transmitter, receiver, and dual-coil combination need to be moved as a whole along the survey line. The weight and volume of mobile devices are very large, requiring high labor and transportation costs, and it is difficult to complete construction in some uneven areas. To overcome the above defects, a nondestructive TEM system with variable offsets is proposed, the instrument design of which is different from traditional equipment. Figure 1b shows the schematic of the proposed TEM system with an MDS for near-surface detection. The TX submodule includes the BCPPS and the TX coil, which generates large trapezoidal current pulses and diffuses a strong primary field to the shallow surface. The corresponding RX submodule includes a receiver and the RX coils, which are carried on a portable vehicle for rapid detection.

Both the BCPPS and the receiver are operated and monitored remotely using wireless communication modules and a control system with a standard software interface. The TX time period of current pulses and the sampling time of induced voltages are synchronized through the world time of the global position system (GPS). Meanwhile, the real-time kinematic (RTK) module will record the position information of the two separated units and upload it to the control software for real-time mapping.

When the TX coil produces a fixed primary field and the RX module movably scans the near surface in a certain range, underground conductors with different resistivities induce voltages in the RX coils due to the eddy current effect after the exciting current pulses are turned off. The three-component inductive coils are arranged in orthogonal order to reflect the variation of the magnetic fields in different directions. The anomaly locations can then be quickly found through data processing and imaging using the system software.

### 2.2. Response Analysis

**Figure 2:**
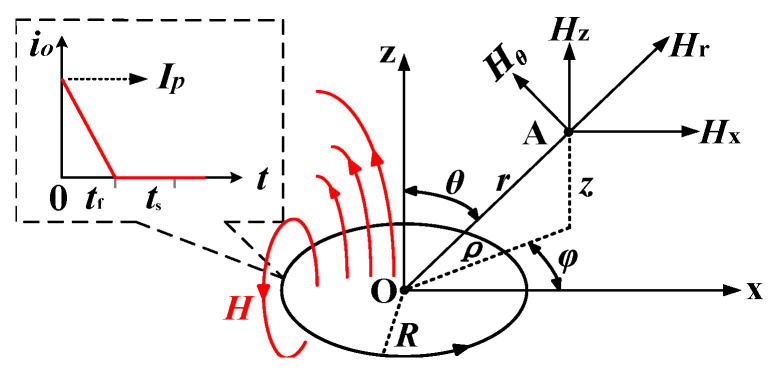
Magnetic field components in a dipole field coordinate system for a typical vertical MDS.

Figure 2 depicts the magnetic field components in the dipole coordinate system for a typical vertical MDS.

In light of the findings presented in Literature 6 and depicted in Figure 2, the magnetic field components along the four directions in the dipole field coordinate system can be expressed as

(1) $$\left\{\begin{array}{l} H_{r}=\frac{M}{4\pi \mu_{0}}\frac{2\cos\theta }{r^{3}}\\ H_{\theta }=-\frac{M}{4\pi \mu_{0}}\frac{\sin\theta }{r^{3}}\\ H_{x}=\frac{M}{4\pi \mu_{0}}\frac{3xz}{{\left(\rho^{2}+z^{2}\right)}^{5/2}}\\ H_{z}=\frac{M}{4\pi \mu_{0}}\frac{\left(2z^{2}-x^{2}\right)}{{\left(\rho^{2}+z^{2}\right)}^{5/2}} \end{array}\right.$$

When φ=0 and the observation point is in the plane of the loop (*z* = 0); the *z*-component of the primary field can be simplified as

(2) $$H_{z}=-\frac{M}{4\pi \mu_{0}r^{3}}=-\frac{NIR^{2}}{4\mu_{0}r^{3}}$$

Equation (2) shows that regular magnetic fields are built up in the surrounding space when the time-varying current flows through the exciting coil placed horizontally on the ground. Increasing the TX magnetic moment *M* enhances the primary field outside the MDS.

**Figure 3:**
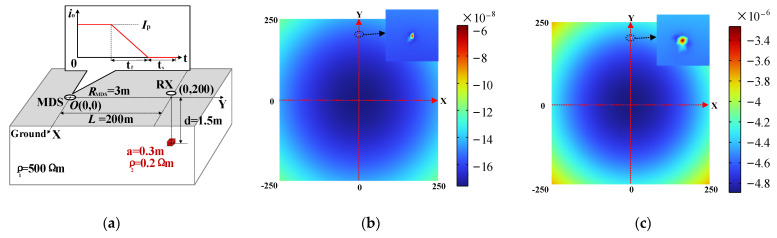
Geoelectric model and finite element simulation results of MDS detection when $t_{s}=200\;\mu s$: (**a**) Three-dimensional geoelectric model and simulation parameters. (**b**) Field distribution Hz (A/m) with $I_{p}=20\;A$ and $t_{f}=200\;\mu s$. (**c**) Field distribution Hz (A/m) with $I_{p}=300\;A$ and $t_{f}=400\;\mu s$.

To ascertain the viability of the proposed detection system, COMSOL (V5.4) simulations were conducted to model the system’s response signal. The geoelectric model is shown in Figure 3a, in which a metal cube with a side length of 0.3 m is buried 200 m away from the MDS on the *y*-axis. The comparison results of the finite element simulation with different MDS parameters are shown in Figure 3b,c. The parameters in Figure 3a are set according to the scheme in the literature [26], and the parameters in Figure 3b are set according to the target parameters of this paper. It can be seen that the larger and steeper current pulses in MDS can excite a stronger secondary field response outside the loop so that the magnetic field above the low-resistivity anomaly shows a greater relative difference from other locations.

Finally, the transient response of nonmagnetic half-space surfaces needs to be further discussed. To simplify the analysis, consider the induced electromagnetic force (EMF) *u_z_* of the *Z* direction. It is shown below that *u_z_* is critical in the subsequent data acquisition and position mapping. This induced EMF generated by the RX coil at a distance *r* from MDS can be expressed as [7].

(3) $$u_{z}=-\alpha S_{r}\mu_{0}\frac{\partial H_{z}}{\partial t}=\frac{\mu_{0}S_{r}I}{4Nt_{f}R}\;\int_{0}^{\infty }G(\lambda ,t)J_{0}(r\lambda )\lambda^{2}d\lambda$$

where *α* is the transition coefficient, *S_r_* is the effective area of the RX coil, and *t_f_* is the falling time of the current. *J*_0_ denotes the zero-order Bessel function [7]. *G* (*λ*, *t*) is a function of the variable *λ* and the observation time *t*; moreover, it is also related to the resistivity and thickness of the formation and can be obtained using the inverse Laplace transform [25]. It can be seen from Equation (3) that larger peak value *I* and shorter falling time *t_f_* of the TX current contribute to a higher response amplitude. The resistivity difference of the underground medium changes the magnetic field above it; this is further reflected by the induced voltage in the RX coils.

## 3. BCPPS and New Charging Strategy

According to the physical model established in Section 3, an increase in distance between the RX module and the MDS rapidly weakens the magnetic intensity. As a result, when the observation device is far from the TX coil, the voltage induced by the secondary field may decay to a level less than that of the ambient electromagnetic noise. This makes it difficult to extract a valid signal for further processing. A solution may be to increase the intensity of the primary field generated using the MDS to strengthen the secondary field at the observation point a distance away.

To achieve this goal, it is required to have a BCPPS that can generate a large current pulse in a high-inductive TX coil [19]. In addition, a flat-top, sharply rising edge and a highly linear falling edge are three distinct features of the current pulse that would have positive effects on the measured response [27].

**Figure 4:**
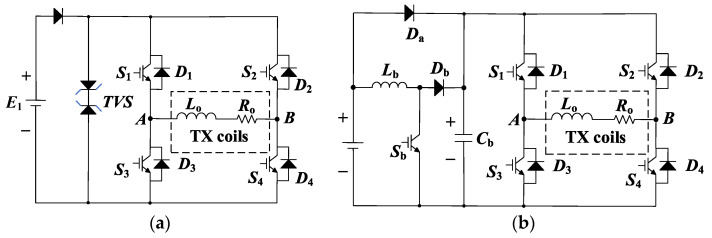
Conventional BCPPS for ground TEM detection: (**a**) Clamping topology with TVS. (**b**) Steep pulse current source with boost topology.

Two conventional BCPPS topologies for ground TEM detection have been proposed for such purpose. One is the clamping scheme using TVS [28] and another is the steep pulse current source scheme [26]. The respective circuit diagrams are shown in Figure 4. The former exhibits a slowly rising edge because the high voltage clamping only occurs during the falling of the pulse [28]. This limits the repeat frequency of the current pulses and the detection efficiency. Moreover, the heating of TVS during clamping is a problem that increases the failure risk along with the operating time. The steep pulse current source scheme employs a boost module to supplement the energy gap and improve the edge steepness of the pulse. However, low charging efficiency makes it difficult to increase the peak value and edge steepness [26]. The multi-pulse charging process also causes considerable noise during the data measurement process [29,30].

### 3.1. Proposed BCPPS

**Figure 5:**
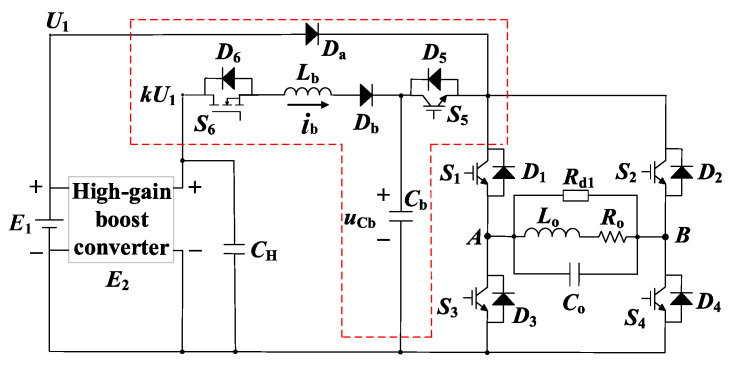
Proposed BCPPS for MDS in the ground TEM detection.

This paper develops a new BCPPS for the TEM detection system using MDS. Figure 5 depicts such a circuit diagram. The source part includes a conventional low-voltage DC power supply *E*_1_ and a high-gain boost converter *E*_2_, which supplies the voltage *kU*_1_. The converter is paralleled with a capacitor bank *C*_H_ to form the constant voltage source. The most critical part of the proposed BCPPS is the resonant charging module, as shown by the red dashed block in Figure 5. It consists of two switches, *S*_5_ and *S*_6_, and an energy storage capacitor, *C_b_*. The resonant charging module performs the energy storage, clamping control, and power switching. It fully charges *C_b_* in the late turn-off period to realize the high-current pulse and enable low-interference output for the MDS.

The proposed BCPPS also contains an H-bridge inverter, which ensures the alternating outputs of current pulses. The TX coil is modeled using the inductance *L_o_*, the resistance *R_o_*, and the stray capacitance *C_o_*. A damping resistor *R*_d1_ is connected in parallel at the terminals of the coil to overcome the underdamped oscillation of the current pulse caused by *C_o_*. The selection of *R_d_*_1_ has been described in [28]. For simplicity, only *L_o_* and *R_o_* of the coil are considered in the analysis. Since *R_d_*_1_ is very large, around several hundred ohms, neglecting *C_o_* does not affect the analysis result in the steady state.

**Figure 6:**
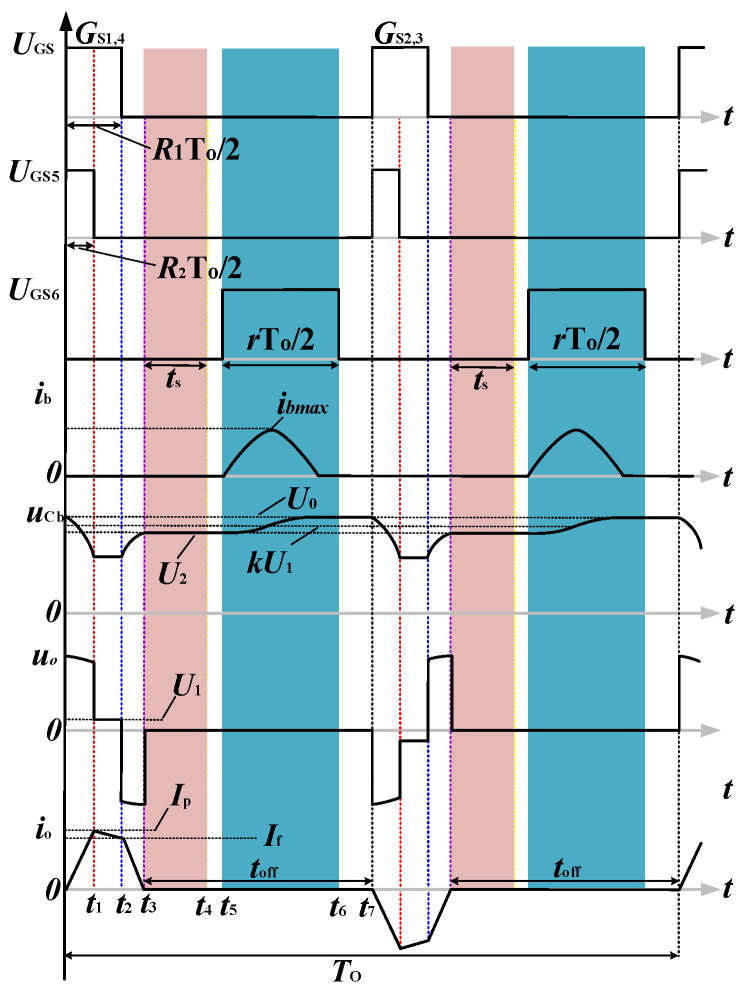
Waveforms of critical elements for the steady state operation of the proposed BCPPS circuit in Figure 5. Red shade: data measuring window. Blue shade: capacitor *C_b_* charging window.

Figure 6 illustrates the waveforms of the voltages and currents of the proposed BCPPS. The positive and the negative current pulse cycles are shown. The analysis below only describes the positive half cycle because the operation principle of the negative cycle is the same as that of the positive one. Current *i_o_* denotes the current pulse generated by the TX coil.

The several variables used in Figure 6 are described as follows. *T_o_* denotes the period of the bipolar current pulse. The duty cycles of the switches from *S*_1_ to *S*_4_ are *R*_1/2_, and the duty cycle of *S*_5_ is *R*_2_; that of *S*_6_ is *r*. The time frame of half the pulse cycle is partitioned into time intervals [0, *t*_1_], [*t*_1_, *t*_2_], [*t*_2_, *t*_3_], and [*t*_3_, *t*_7_]. The first interval denotes the pulse rising period, the second is the flat-top period of the pulse, the third is the pulse falling period, and the fourth is the pulse turn-off period. Critical parameters include the pulse rise time *t_r_* = *t*_1_, the pulse falling time *t_f_* = *t*_3_ − *t*_2_, the flat-top duration *t_FT_* = *t*_2_ − *t*_1_, the current turn-off duration *t_off_* = *t*_7_ − *t*_3_, and the turn-on duration of the switch *S*_6,_ *t_b_* = *t*_6_ − *t*_5_. These parameters can be expressed, in terms of *T_o_* and the duty cycles, as

(4) $$\left\{\begin{array}{l} t_{r}=\frac{R_{2}T_{o}}{2}\\ t_{FT}=\frac{(R_{1}-R_{2})T_{o}}{2}\\ t_{off}=\frac{(1-R_{1})T_{o}}{2}-t_{f}\\ t_{b}=\frac{rT_{o}}{2} \end{array}\right.$$

### 3.2. Operational Principles

Four operation modes of BCPPS are considered. Mode 1 is the pulse rising period *t*_r_. Mode 2 is the pulse flat-top period *t_FT_*. Mode 3 is the pulse falling period *t_f_* = *t*_3_ − *t*_2_. Mode 4 is the pulse turn-off period, where the turn-off time is *t_off_*. It includes two parts. The first part appears at the early stage of this period, [*t*_3_, *t*_4_]. It is called the measuring window and is marked by the red shape in Figure 6. The second part appears in the late stage, [*t*_5_, *t*_6_]. It is called the charging window and is marked in blue in Figure 6.

**Figure 7:**
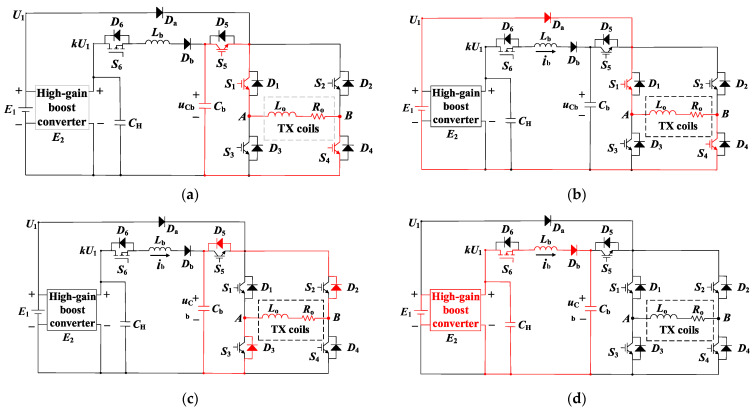
Four operation topologies of the proposed BCPPS, indicated by the red loops: (**a**) Mode 1. (**b**) Mode 2. (**c**) Mode 3. (**d**) Mode 4.

The corresponding circuit configurations of these four operation modes are shown in Figure 7a–d. Detailed operation principles are explained below.

#### 3.2.1. Mode 1

During the *t_r_* time period of the positive current pulse, switches *S*_1_, *S*_4_, and *S*_5_ are turned on, while *S*_2_, *S*_3_, and *S*_6_ are off. Note that both the rising time and the steep rise of the current pulse depend on the turn-on interval of *S*_5_. It can be seen from Figure 7a that *C_b_*, *S*_5_, *S*_1_, *L_o_*, *R_o_*, and *S*_4_ form a resonant loop. The current in the TX coil rises rapidly to its peak value *I_p_*. The dynamic of the current pulse is governed by

(5) $$L_{o}C_{b}\frac{d^{2}i_{o}(t)}{dt^{2}}+R_{o}C_{b}\frac{di_{o}(t)}{dt}+i_{o}(t)=0,t\in [0,t_{1}]$$

The values of electrical elements should be selected to satisfy the following relationship

(6) $${\left(R_{o}C_{b}\right)}^{2}-4L_{o}C_{b}<0$$

Suppose that the initial voltage of the capacitor *C_b_* is *U*_0_. Solving Equation (4) yields the maximum instantaneous current *I_p_* = *i_o_* (*t*_1_), which is expressed as

(7) $$I_{p}=\frac{U_{0}}{\mu_{1}L_{o}}e^{-\frac{\delta_{1}R_{1}T_{o}}{2}}\sin(\frac{\mu_{1}R_{2}T_{o}}{2})$$

The parameters *μ*_1_ and *δ*_1_ are

(8) $$\left\{\begin{array}{l} \mu_{1}=\frac{\sqrt{4L_{o}C_{b}-{\left(R_{o}C_{b}\right)}^{2}}}{2L_{o}C_{b}}\\ \delta_{1}=\frac{R_{o}}{2L_{o}} \end{array}\right.$$

#### 3.2.2. Mode 2

This is the flat-top stage of the current pulse. It can be seen from Figure 7b that switches *S*_1_ and *S*_4_ remain on while *S*_5_ is turned off. The supply channel between *C_b_* and the H-bridge inverter is cut off so that diode *D*_5_ is on. Now, the TX coil is powered by *E*_1_. During this interval, *S*_1_ and *S*_4_ are the only switches conducting. By neglecting the voltage drop of the diodes and switches, the current is governed by

(9) $$\left\{\begin{array}{l} R_{o}i_{o}(t)+L_{o}\frac{di_{o}(t)}{dt}=U_{1},t\in [t_{1},t_{2}]\\ i_{o}(t_{1})=I_{p} \end{array}\right.$$

Then, the ultimate instantaneous current *I_f_* can be obtained

(10) $$I_{f}=\frac{U_{1}}{R_{o}}+(I_{p}-\frac{U_{1}}{R_{o}})e^{-\delta_{1}(R_{1}-R_{2})T_{o}}$$

In fact, the flat-top current is not flat. It falls approximately in a linear fashion with the slope

(11) $$\gamma =\frac{2(1-e^{-\delta_{1}(R_{1}-R_{2})T_{o}})}{(R_{1}-R_{2})T_{o}}(\frac{U_{1}}{R_{o}}-I_{p})$$

The pulse is of a trapezoidal type (flat top) only when *U*_1_ = *R_o_I_p_*. Thus, in actual detection applications, the proper selection of *U*_1_ and *t_FT_* is critical to obtain an accurate secondary field signal.

#### 3.2.3. Mode 3

The current pulse falling edge. In this period, switches *S*_1_ and *S*_4_ are turned off while their opposing freewheeling diodes *D*_2_ and *D*_3_ are forward-biased. When the current in the TX coil decreases to zero, all remaining energy is transferred to the energy-storage capacitor. Assuming that the voltage of *C_b_* at the end of this stage is *U*_2_, the second-order differential Equation (4) in mode 1 can also apply to this stage:

(12) $$U_{2}=\mu_{1}L_{o}I_{f}e^{\delta_{1}t_{f}}/\sin(\mu_{1}t_{f})$$

#### 3.2.4. Mode 4

Regarding the turn-off period of the current pulse, i.e., during this period, *i_o_* = 0 *i_AB_* = 0. This mode performs two critical operations. One is to record the induced voltage. This operation occurs at the early stage of this period. It is called the measuring window and is marked in red in Figure 6. Another is to charge the high-voltage capacitor *C_b_* for the generation of the next current pulse. This operation occurs in the later part of this period. It is called the charging window, and it is marked in blue in Figure 6.

In the measuring window, switches *S*_1_ to *S*_6_ are all turned off and the high-gain boost converter is in the no-load state. The switching noise of BCPPS is the lowest. This creates a low-interference period for the receiver to sample the induced voltages. On the other hand, the early secondary field signal is, relatively, more sensitive to the underground anomalies because of the separated MDS, particularly when the distance between the TX loop and RX coils is tens of meters away. In this case, the BCPPS is quiet during the measuring time. This is good for obtaining accurate detection results.

**Figure 8:**
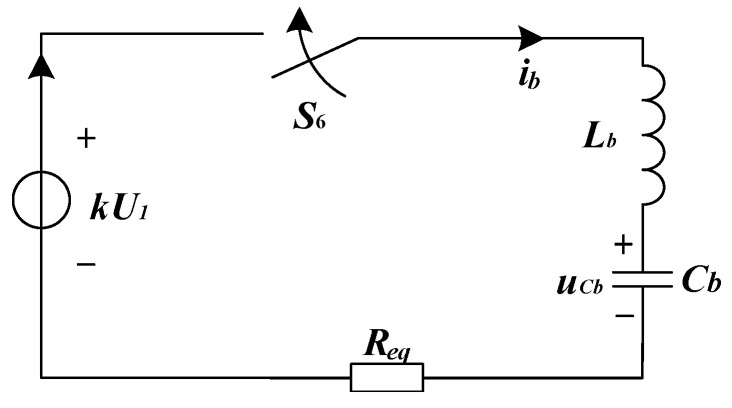
Equivalent circuit of the resonant charging loop.

In the charging window, *S*_6_ is turned on and other switches remain off. It can be seen from Figure 7d that the high-gain boost converter, *E*_2_, *L_b_*, and *C_b_* form a resonant loop to charge the capacitor voltage. The equivalent circuit of the resonant charging loop is shown in Figure 8.

*R*_eq_ represents the equivalent resistance of switch *S*_6_, inductor *L_b_*, and capacitor *C_b_*. The capacitor voltage is governed by

(13) $$L_{b}C_{b}\frac{d^{2}u_{Cb}(t)}{dt^{2}}+R_{eq}C_{b}\frac{du_{Cb}(t)}{dt}+u_{Cb}(t)=kU_{1},$$

for $t\in [t_{5},t_{6}]$. The current through the inductor can be obtained by

(14) $$i_{b}(t)=C_{b}\frac{du_{Cb}(t)}{dt}$$

The parameters should satisfy the following relationship,

(15) $${\left(R_{eq}C_{b}\right)}^{2}-4L_{b}C_{b}<0$$

The capacitor voltage *u_Cb_* can be obtained by

(16) $$u_{Cb}(t)=kU_{1}+(U_{2}-kU_{1})\frac{\sqrt{\mu_{2}{}^{2}+\delta_{2}{}^{2}}}{\mu_{2}}e^{-\delta_{2}(t-t_{5})}\sin[\mu_{2}(t-t_{5})+\theta_{2}]$$

and the charging current *i_b_* by

(17) $$i_{b}(t)=\frac{kU_{1}-U_{2}}{\mu_{2}L_{b}}e^{-\delta_{2}(t-t_{5})}\sin[\mu_{2}(t-t_{5})]$$

Parameters *μ*_2_, *δ*_2_, and *θ*_2_ are

(18) $$\left\{\begin{array}{l} \mu_{2}=\frac{\sqrt{4L_{b}C_{b}-{\left(R_{eq}C_{b}\right)}^{2}}}{2L_{b}C_{b}}\\ \delta_{2}=\frac{R_{eq}}{2L_{b}}\\ \theta_{2}=\arctan(\frac{\mu_{2}}{\delta_{2}}) \end{array}\right.$$

It follows from Equation (16) that the effective charging time is $t_{c}=\pi /\mu_{2}$ and, at $t=t_{5}+\theta_{2}/\mu_{2}$, the charging current $i_{b}$ reaches its peak value

(19) $$I_{b,\max}=\frac{kU_{1}-U_{2}}{\mu_{2}L_{b}}e^{-\frac{\delta_{2}\theta_{2}}{\mu_{2}}}\sin(\theta_{2})$$

It is required that the maximum instantaneous output power of the high-gain boost converter *E*_2_ should exceed *kU*_1_*I_b,max_*. Moreover, the saturation current of the inductor *L_b_* must be larger than *I_b,max_*. Since the resonant process needs to be completed within the on-time *t_b_* of *S*_6_, the value of *L_b_* should be selected in the range

(20) $$\frac{t_{b}(t_{b}-\sqrt{t_{b}{}^{2}-\pi^{2}R_{eq}{}^{2}C_{b}{}^{2}})}{2\pi^{2}C_{b}}\leq L_{b}\leq \frac{t_{b}(t_{b}+\sqrt{t_{b}{}^{2}-\pi^{2}R_{eq}{}^{2}C_{b}{}^{2}})}{2\pi^{2}C_{b}}$$

Another advantage of this design is that the charging current would drop to zero before S_6_ is turned off. Thus, there is almost no turn-off loss during the switch. When the resonant charging is completed, the capacitor voltage *u_Cb_* is raised to

(21) $$U_{0}=kU_{1}+(kU_{1}-U_{2})\frac{\sqrt{\mu_{2}{}^{2}+\delta_{2}{}^{2}}}{\mu_{2}}e^{\frac{-\pi \delta_{2}}{\mu_{2}}}\sin(\theta_{2})$$

The above derivation is the basis of the capacitance and voltage selection for *C_b_*. It is also known that the clamping voltage between the terminals of the TX loop is determined using the gain of the converter, the parameters of passive devices, and the conducting time of the switches in the proposed BCPPS.

Considering that the energy storage capacitor can still rely on the energy feedback of the load inductor to raise the voltage in the case of non-resonant charging, it is obvious that *L_b_* can only participate in the replenishment process when the charging voltage is higher than the upper limit of the clamp voltage in the case of single-wave charging. The minimum design value of the converter gain *k* can be obtained as follows:

(22) $$k_{\min}=\frac{\mu_{0}}{2\delta_{0}}\cdot \frac{1-e^{(R_{2}-R_{1})\delta_{0}T_{0}}}{\sqrt{1-e^{-R_{2}\delta_{0}T_{0}}}-e^{(\frac{R_{2}}{2}-R_{1})\delta_{0}T_{0}}\sin(\frac{\mu_{0}R_{2}T_{o}}{2})}$$

Theoretically, the larger the gain, the more energy will be transferred from the power supply end to the capacitor *C_b_*; however, it is also necessary to consider the current output capacity of the boost converter and the output parallel capacitor. The maximum design value of *k* can be obtained as follows:

(23) $$k_{\max}=\frac{\mu_{0}}{2\delta_{0}}\cdot \frac{1-e^{(R_{2}-R_{1})\delta_{0}T_{0}}}{\sqrt{1-e^{-R_{2}\delta_{0}T_{0}}}-e^{(\frac{R_{2}}{2}-R_{1})\delta_{0}T_{0}}\sin(\frac{\mu_{0}R_{2}T_{o}}{2})}+\frac{\mu_{2}L_{b}I_{M}e^{\delta_{2}\theta_{2}/\mu_{2}}}{U_{S}\sin\theta_{2}}$$

where *I_M_* is the peak output current of *E*_2_ and *C_H_*.

Compared with other charging schemes such as the current-limiting resistor, it not only achieves high efficiency and low-loss charging but also greatly reduces the source’s interference in the sampling interval of the RX signal.

## 4. System Design

**Figure 9:**
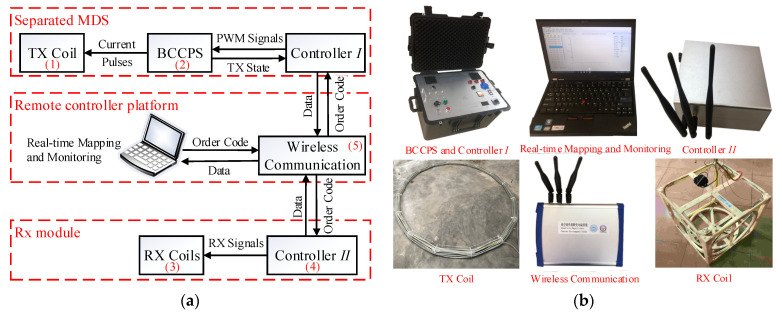
Composition of the proposed electromagnetic measurement system with separated MDS: (**a**) Functional block diagram of the system. (**b**) Physical photos of the system.

This section describes the design considerations of the proposed MDS-TEM measurement system. The main units include the MDS, data acquisition and processing, and system integration. Figure 9 shows the hardware of these components. The numbers in the parentheses of the components correspond to those in Figure 1b.

### 4.1. Design of MDS

The MDS module consists of TX coils and the new BCPPS. BCPPS is designed to supply high-current pulses to the TX coil with a peak magnitude exceeding 300 A. The circuit diagram is shown in Figure 4 and the hardware is shown in Figure 8. Switches *S*_1_ to *S*_5_ are IGBT switches needed for the high-current pulse, and *S*_6_ is a MOSFET switch, which has low conduction loss and is more efficient for the capacitor charging process. A boost converter with an adjustable gain of 10 to 40 for output voltage is used to charge the capacitor in the late turn-off period of the current pulse. The magnitude of a current pulse can be adjusted by varying the boost gain. In field detection, the gain *k* can be selected according to the power provided by *E*_1_ and the size of the to-be-detected area.

The MDS module can be set at one location and does not need to move around during each TEM detection process. The TX coil is wound by 40 turns of the metal wire, and its diameter is about two meters. The MDS (coils plus BCPPS) weighs around 30 kg.

### 4.2. Design for Data Receiving

**Figure 10:**
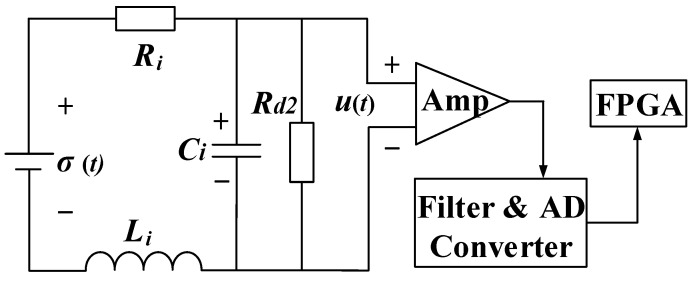
RX signal acquisition and preliminary processing.

In the conventionally integrated TX-RX configuration, the coupling effect is one of the design considerations [10,11,13]. This effect is no longer a concern in the proposed MDS-TEM system because RX coils and the TX loop are separate and far away from each other. The scanning platform only contains the RX module, which includes RX coils and the controller for sampling and data transmission. This greatly simplifies the design process. This structure facilitates the voltage induction from three orthogonal planes because the magnetic fields above the detection area are oriented in multiple directions. Figure 10 shows the diagram of RX signal acquisition and the processing unit.

In Figure 10, *σ*(*t*) is the induced EMF, *R*_d2_ is the damping resistance, and *R_i_*, *L_i_*, and *C_i_* are the internal resistance, inductance, and distributed capacitance of the RX coil, respectively. The distributed capacitance *C_i_* is lowered using the piecewise structure and fillers in a multi-layer winding. This expanded the −3 dB bandwidth of the coil. The transfer function from *σ*(*t*) to *u*(*t*) can be derived as

(24) $$H(s)=\frac{R_{d2}}{s^{2}L_{i}C_{i}R_{d2}+s(L_{i}+C_{i}R_{i}R_{d2})+R_{i}+R_{d2}}$$

The amplified analog signal is then processed using a band-pass filter and a high-precision AD converter. When digitized signals are transmitted to FPGA, the induced voltages during the early turn-off period of the current pulse are extracted and uploaded to the control platform via wireless communication.

### 4.3. System Integration

The proposed MDS-TEM also contains a remote control platform. One controller is designed specifically for BCPPS. Another is to sample the induced voltage in the RX module. Both controllers are embedded in the real-time kinematic (RTK) module. The functions are used to record the positions of the MDS and RX for data normalization and to upload to the control software (V1.1.2) for real-time mapping. RTK also generates the GPS pulses for the synchronization of these two controllers. The time difference between these two modules is limited to 100 ns by a cycle correction algorithm to contain the instability of the crystal oscillator in FPGA.

A 2.4G wireless communication module is developed to receive and send data between controllers and a PC so that BCPPS can be controlled by a PC from a distance (hundreds of meters away). The module also monitors the charging voltage and current pulse. The induced voltage of the RX coils can be uploaded remotely and used for real-time mapping. This paves the way for small drones or unmanned vehicles to be deployed to scan the target areas and collect data.

## 5. Experimental Results

This section presents the experimental results to verify the effectiveness of the proposed MDS-TEM system. Three aspects are evaluated. First, current pulses generated by the proposed BCPPS are compared with those generated using conventional BCPPS circuits to show the benefits of the proposed BCPPS circuit. Second, the induced voltages of the RX module are presented to indicate that the detection of an anomaly is possible. Third, field test results are shown to demonstrate the detection effectiveness of the proposed MDS-TEM system.

### 5.1. Performance of the BCPPS

**Figure 11:**
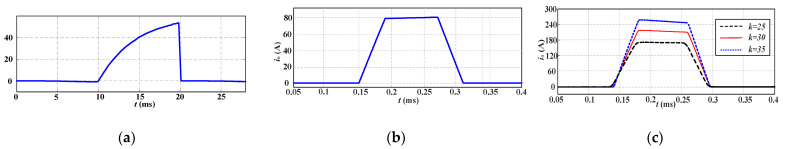
Current pulse waveforms generated using various BCPPS circuits: (**a**) Clamping scheme with TVS. (**b**) Steep pulse current source with boost module. (**c**) Proposed topology for MDS with different converter gains.

Table 1 lists the main parameters of the MDS and BCPPS. Figure 11 shows the current pulses generated using three BCPPS circuits.

Figure 11a shows the current pulse generated using the clamping scheme, exhibiting a steep falling edge and a very slowly rising edge; moreover, the magnitude is less than 60 A. The current pulse shown in Figure 11b is generated using the boost topology, displaying flat top, linear and fast rising and falling edges, and a magnitude of up to 80 A. Figure 11c shows the current pulses generated using the proposed method with adjustable voltage gains. The pulses exhibit linear and steep rising and falling edges with a magnitude ranging from 160 A to 270 A. For example, with a charging voltage of 920 V, the peak current pulse reaches 270 A in 0.4 ms. This provides a large TX magnetic moment for a magnetic dipole of a 2 m radius and 40 turns of coils. Note that the flat top of the current pulse is not strictly flat, especially when the charging voltage gain is large. The slope of the flat top can be improved by modifying the circuit parameters according to Equation (11).

### 5.2. Field Test

**Figure 12:**
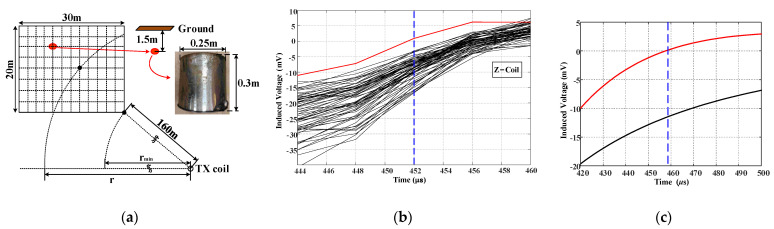
Measured induced voltages of the survey line. The measuring points are located at the intersection of the dotted lines: (**a**) Experimental layout of the detection process. (**b**) Z-coil data during the early turn-off process at measuring points. (**c**) The simulation results for induced voltage in scenarios with and without a metal body.

A designed experiment was carried out in an agricultural area with less electromagnetic interference to evaluate the performance of the proposed system. Figure 12a shows the test arrangement. The field scanned using the RX module was a 30 m × 20 m rectangular area that was partitioned by dashed lines into small squares with a size of 2.5 m × 2.5 m. The intersection points of the dashed lines are the measuring points at which the RX scanning is taken. The scanned area is at least 160 m away from the center of the TX coil.

Suppose that a metal bucket was buried 1.5 m underground at the intersection point marked by the red dot in Figure 12a as the anomalous object. The diameter of the cylindrical bucket is 0.25 m, and the height is 0.3 m. The peak current of the pulse was 270 A after adjusting the converter’s gain. The region was scanned continuously using the RX module. The measured waveforms of the induced voltages at all measuring points were extracted using RTK coordinates. These waveforms were used to locate anomalous objects.

Figure 12b shows the induced voltages recorded at Z-coil during the early stage of the turn-off process. The red curve is the voltage measured directly above the anomalous body, as shown by the red dot in Figure 12a. The black curves are voltages recorded at other measuring points. The red curve of the voltage in Figure 12b shows a smaller amplitude than the black curves, and it changes the polarity from negative to positive much earlier compared with the black curves. This may be because the eddy current generated by the underground metal body slows down the attenuation of the magnetic field. This phenomenon is consistent with the theoretical analysis presented in Section 2. Additionally, Figure 12c displays the simulated results of an induced voltage with (red curve) and without (black curve) a metallic body. The zero-crossing characteristics when a metal body is present align with the experiment data, with the zero-crossing points occurring earlier than in the scenarios without a metal body.

**Figure 13:**
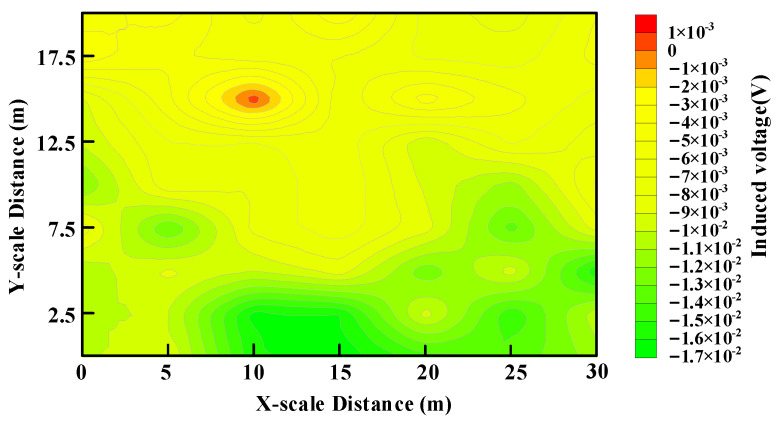
Position imaging of the induced voltage scanning.

Figure 13 shows the contour map of the induced voltage scan. It represents the induced voltage signal at 452 μs following the commencement of the transmitting current turn-off. The horizontal axis and vertical axis coordinates correspond to the scanned area of Figure 12. In Figure 13, the areas in red denote the positively induced voltages, and the remaining areas denote the negative voltages. The polarity change of the induced voltage is mainly due to the stronger eddy current effect caused by the underground, low-resistant metal body than that of the soil. This polarity change phenomenon can be used to locate shallow and underground low-resistant anomalous objects. Thus, it is concluded that the area in red is where the anomalous body is buried below. Figure 13 can be obtained immediately after the scanning of the target region because the induced voltage is extracted directly. This result shows that the proposed MDS-TEM system provides an alternative means for near-surface detection in addition to the conventional RX-TX-integrated systems [10,13].

Table 2 shows the operation parameters of the systems in [10,13] and that of the proposed system. Table 2 shows that the proposed system offers a much higher TX peak magnetic moment and smaller RX size and weight than the other two TEM systems. The length of the mobile acquisition device is less than 1.0 m, and the weight is less than 3.0 kg. Separate and small RX modules allow for more flexibility in operation and offer much larger surveying areas than the TX-RX-integrated systems.

## 6. Conclusions

This paper presents an MDS-TEM system with separate TX and RX configurations. This MDS-TEM system has a fixed excitation source, a magnetic dipole source, and a mobile RX module that is small, lightweight, and remotely controlled. A new BCPPS design is also proposed to solve the challenge that the primary field may not be large enough to produce a strong secondary field a distance away from the TX module so that valid induced voltages can be obtained. The critical unit of this BCPPS is the resonant charging module, which performs energy storage, clamping control, and power switching. It provides resonant charging in the late turn-off period to realize the high-current pulse and low-interference output for the MDS. Detailed operation principles of BCPPS have been described, and performance comparisons with conventional BCPPS circuits were presented. Compared to other TEM systems, the proposed system, due to its use of separate transmitting and receiving coils, faces a limitation where the primary field generated by the transmitting coil travels a greater distance to the anomaly targets than in other TEM systems. This limits the system’s maximum detection depth and range. We address this issue by designing a resonant dual-wave supplementary energy circuit to increase the magnetic moment, thereby compensating for deficiencies in detection depth and range. Additionally, due to structural differences in the system, the correction and inversion of signals received by the coils present new challenges, which we are actively working to resolve.

This experiment has been carried out to show that the proposed TEM detection system provides satisfactory near-surface detection results while the RX module is positioned at a 200 m horizontal distance away from the TX module. The new BCPPS can provide a stable TX peak current of up to 300 A and a peak magnetic moment of 3.75 × 10^4^ Am^2^. Mobile RX scanning greatly reduces shallow detection costs and improves survey efficiency.

## Figures and Tables

**Table 1:** Parameter values of MDS.

	Parameters	Value
BCPPS	Supply voltage (*U*_1_)	24 V
	Gain of the converter (*K*)	10~40
	Resonant inductor (*L_b_*)	0.5 mH
	Saturation current of *L_b_* (*i_bmax_*)	10 A
	Pulse repetition frequency (*f_t_*)	50 Hz
	Rising time of pulse (*t_r_*)	0.4 ms
	Flat-top time of pulse (*t_p_*)	0.8 ms
TX loop	Diameter of the TX coil (*D_t_*)	2 m
	Number of turns (*N_t_*)	40
	Total TX area (*S_t_*)	125 m^2^
	Coil inductance (*L_o_*)	1.5 mH
	Coil resistance (*R_o_*)	0.25 Ω
	Damping resistance (*R_d_*_1_)	800 Ω
	TX peak moment (*M_t_*)	37,500 Am^2^

**Table 2:** Comparison of three TEM systems for near-surface detection.

Scheme	TX Area (m^2^)	TX Peak Current (A)	TX Peak Moment (Am^2^)	Mobile Device		Mode
				L (m)	W (kg)	
[10]	11.3	70	791	3–8	35	Uncontinuous
[13]	8	30	240	14	>70	Continuous
Proposed	125	300	37,500	<1	3	Continuous

## Data Availability

The data that support the findings of this study are available from the corresponding author upon reasonable request.
